# Predictors of HBeAg status and hepatitis B viraemia in HIV-infected patients with chronic hepatitis B in the HAART era in Brazil

**DOI:** 10.1186/1471-2334-11-247

**Published:** 2011-09-20

**Authors:** Maria Cassia Mendes-Correa, João RR Pinho, Michele S Gomes-Gouvea, Adriana C da Silva, Cristina F Guastini, Luiz G Martins, Andréa G Leite, Mariliza H Silva, Reinaldo J Gianini, David E Uip

**Affiliations:** 1 Infectious Diseases Research Unit, ABC Medical School, Santo André, Brazil; 2 Department of Infectious Diseases Medical School, University of São Paulo, São Paulo, Brazil; 3 Tropical Medicine Institute and Department of Gastroenterology, Medical School, University of São Paulo, São Paulo, Brazil; 4 AIDS Outpatient Clinic, São Bernardo do Campo, São Paulo, Brazil; 5 Laboratory of Medical Investigation in Epidemiology and Statistics, Medical School, University of São Paulo, São Paulo, Brazil

**Keywords:** hepatitis B virus, HIV, virological outcome, tenofovir

## Abstract

**Background:**

HBV-HIV co-infection is associated with an increased liver-related morbidity and mortality. However, little is known about the natural history of chronic hepatitis B in HIV-infected individuals under highly active antiretroviral therapy (HAART) receiving at least one of the two drugs that also affect HBV (TDF and LAM). Information about HBeAg status and HBV viremia in HIV/HBV co-infected patients is scarce. The objective of this study was to search for clinical and virological variables associated with HBeAg status and HBV viremia in patients of an HIV/HBV co-infected cohort.

**Methods:**

A retrospective cross-sectional study was performed, of HBsAg-positive HIV-infected patients in treatment between 1994 and 2007 in two AIDS outpatient clinics located in the São Paulo metropolitan area, Brazil. The baseline data were age, sex, CD4 T+ cell count, ALT level, HIV and HBV viral load, HBV genotype, and duration of antiretroviral use. The variables associated to HBeAg status and HBV viremia were assessed using logistic regression.

**Results:**

A total of 86 HBsAg patients were included in the study. Of these, 48 (56%) were using combination therapy that included lamivudine (LAM) and tenofovir (TDF), 31 (36%) were using LAM monotherapy, and 7 patients had no previous use of either one. Duration of use of TDF and LAM varied from 4 to 21 and 7 to 144 months, respectively. A total of 42 (48. 9%) patients were HBeAg positive and 44 (51. 1%) were HBeAg negative. The multivariate analysis revealed that the use of TDF for longer than 12 months was associated with undetectable HBV DNA viral load (serum HBV DNA level < 60 UI/ml) (*p *= 0. 047). HBeAg positivity was associated with HBV DNA > 60 UI/ml (p = 0. 001) and ALT levels above normality (*p *= 0. 038).

**Conclusion:**

Prolonged use of TDF containing HAART is associated with undetectable HBV DNA viral load. HBeAg positivity is associated with HBV viremia and increased ALT levels.

## Background

Hepatitis B virus (HBV) and human immunodeficiency virus (HIV) co-infection is a frequent event because both viruses share the same routes of transmission. Studies conducted in North America, Europe, and Australia have shown that between 5% and 10% of all HIV-infected patients are also infected with HBV [1]. In Brazil, this prevalence ranges from 1. 6% to 8. 5% [2-8].

HIV infection has a negative impact in all phases of the natural history of hepatitis B, leading to increased rates of persistent infection, higher HBV DNA levels, and lower rates of hepatitis B and antigen loss [9-11]. Progression of fibrosis, development of cirrhosis, and liver-related mortality are more frequent among HIV-HBV co-infected patients than among individuals infected with either one alone [9-11].

However, it is still unknown what kind of impact long-term use of the highly active antiretroviral therapy (HAART), which includes lamivudine (LAM) and tenofovir (TDF) (two drugs that are also active against HBV), may have on the natural history of chronic hepatitis B in co-infected individuals The impact of the use of antiretroviral therapy in HBeAg status and HBV viremia has rarely been investigated among HIV-infected patients receiving highly active antiretroviral therapy.

Therefore, the aim of the present study was to evaluate clinical and virological variables that may influence HBeAg status and HBV viraemia in a HIV/HBV co-infected cohort followed up in São Paulo Metropolitan area, Brazil, that were under antiretroviral therapy which included anti-HBV drugs, such as lamivudine (LAM) and tenofovir (TDF).

## Methods

### Study Design

A retrospective cross-sectional study was conducted in two AIDS outpatient clinics located in the São Paulo metropolitan area, Brazil. HIV-HBV co-infected patients were included in the study.

### Patients

This retrospective study evaluated the medical records of all HIV patients under treatment between November 1994 and November 2007 in an attempt to identify patients who were also infected with HBsAg. Clinical, demographic, and serological data contained in the medical records of HIV patients who had anti-HIV antibodies confirmed by Western blot and positive HBsAg serum for longer than 6 months were analyzed.

The data collected at the time a serum sample was drawn were: age, sex, CD4 T-cell count, use of antiretroviral therapy, plasma HIV-RNA level, TDF and LAM therapy and time of use of these drugs, HBeAg status, HBV viral load, HBV genotype, presence of hepatitis C infection and hepatitis D super-infection, presence of advanced liver disease, alanine aminotransferase (ALT) level, and compliance with therapy.

Antiretroviral therapy (including TDF and LAM) usage was defined as the use of any antiretroviral medication for longer than 6 months at any time prior to the time a serum sample was drawn. Compliance to therapy was assessed by the assistant physician. Patients were considered compliant when their medical records indicated that they had used at least 80% of the medication prescribed by their doctor.

The Local Ethics Research Boards approved this study these were the "Comissão de Bioética-CoBi, do HCFMUSP" (Ethics Commission-Hospital das Clinicas, Sao Paulo University Medical School) and "Comissão de Ética da FMABC" (Ethics Commission-ABC Foundation Medical School). All patients who participated in the study signed an informed consent form.

### Laboratory Testing

Hepatitis B serological testing was performed according to standard procedures and using licensed assays, including HBsAg, total anti-HBc, anti-HBs, HBeAg, and anti-HBe.

Absolute quantification of HBV DNA was carried out by an in-house real-time polymerase chain reaction (PCR) [12] assay using primers capable of amplifying HBV genotypes A to H (28 samples), or by the commercially available COBAS Amplicor HBV Monitor Test assay (Roche Molecular Systems, Pleasanton, CA, USA) according to the manufacturer's instructions (58 samples), as previously described [13,14].

HBV-positive DNA was further amplified and followed by sequencing of the 737 bp PCR fragment covering amino acids 48 to 288 to determine genotypes and antiviral resistance. We used primers FHBS1, FHBS2, RADE1M, and RADE2M described in previous studies to amplify a larger informative region of the Polymerase gene [15,16].

Hepatitis C virus infection was defined by the presence of anti-HCV antibodies (ELISA, 3rd generation). Current HCV infection was confirmed by PCR.

Hepatitis Delta virus (HDV) co-infection was defined by the presence of positive anti-HDV antibodies by ELISA. Current HDV infection was confirmed by PCR, and HDV genotype was determined by sequencing and phylogenetic analysis, as previously reported [16].

Alanine transaminase (ALT) level was examined at baseline and on at least two separate occasions during the 12 months before baseline. A normal ALT level was considered when at least three consecutive normal ALT results were obtained during a follow-up of 12 months.

### Statistical Analysis

Statistical analyses were carried out using Stata software (Stata Corp., College Station, TX, USA). We initially conducted an analysis of frequency distribution for each selected variable. Continuous parameters were compared between groups using Student's t-test, except for continuous variables without normal distributions or those for small groups, in which case the parameters were compared using the Mann-Whitney U-test. For categorical variables, the Pearson χ^2 ^or Fisher's exact test were used according to group size. Differences were considered statistically significant when *p *< 0. 05. Multivariate logistic regression analysis was performed to examine the association of several variables and HBeAg status and HBV viremia.

## Results

A total of 3,259 patients from the two AIDS outpatient clinics with confirmed HIV infection and serological markers for hepatitis B were included. Among them, 154 (4. 7%) were HBsAg-positive. Of these 154, only 86 for whom blood samples were available were included in the study.

The main characteristics of the study population are shown in Table 1. Most of the patients were males (96. 5%) and the mean age was 42 years. Patients were analyzed according to their risk factors for hepatitis B infection. Sixty-four (74. 4%) were men who had sex with men and 16 (18. 6%) were intravenous drug users. Seventeen patients (19. 7%) presented clinical evidence of advanced liver disease (splenomegaly, ascites, and esophageal varices). Nine patients (10. 4%) were not compliant to the therapy.

**Table 1:** Main baseline characteristics of 86 HIV/AgHBs co-infected patients

Variables	n
Male gender (%)	83 (96. 5%)
Mean age (years)	42 (± 7. 2)
Mode of HIV transmission	
-Men who have unprotected sex with men (%)	64 (74. 4%)
- Exchange of needles among intravenous drug users (%)	16 (18. 6%)
Clinical Variables	
- Advanced hepatic disease	17 (19. 7%)
-Non adherence to treatment	09 (10. 4%)
Treatment Given-HAART	
-Previous use of antiretroviral therapy	81 (94. 1%)
-Patients under LAM + TDF combination therapy	48 (56. 0%)
-Patients under LAM monotherapy	31 (36. 0%)
-Previous use of either LAM or TDF	81 (94. 1%)
- Previous use of LAM	79 (91. 8%)
- Previous use of TDF	48 (55. 8%)
-Time of use of TDF (months)	4-21
-Time of use of LAM (months)	7-144
-Laboratory Variables	
HBeAg positive	42 (48. 9%)
HBV-DNA < 60 UI/mL	50 (58. 1%)
HBV-DNA > 60 UI/mL	34 (39. 5%)
HBV-DNA unknown	02 (2. 32%)
HBV Genotype	
-genotype A	21
-genotype D	05
-genotype G	05
-genotype F	03
-CD4 cell count, median cells/μ1	545 (± 250)
-Baseline HIV RNA < 400 copies/mL	73 (85%)
ALT level, normal	54 (62. 7%)
- ALT level > 1. 5 × ULN†	32 (37. 2%)
-Anti-HCV positive	20 (23. 2%)

-Anti-HDV positive	01 (1. 1%)

ULN†-Upper Limit of Normality; LAM = lamivudine; TDF = tenofovir

Among the 86 patients, 81 (94. 1%) declared previous use of antiretroviral therapy. For the two drugs that also work against HBV (LAM and TDF), 79 (91. 8%) declared previous use of the former and 48 (55. 8%) of the latter. At the baseline, 31 (36%) and 48 (56%) of them were under LAM monotherapy or combination therapy of both drugs, respectively. All patients under TDF monotherapy were changed to combination therapy with LAM. Time of exposure to TDF and LAM varied from 4 to 21 and 7 to 144 months, respectively.

Among the 34 patients with detectable HBV viremia, 5 had never received any anti-retroviral treatment, 29 had used LAM, and 13 had also used TDF (LAM + TDF). Sixteen patients with detectable HBV viremia were on LAM monotherapy and among them, eight patients presented LAM-resistant mutations. Thirteen patients under LAM + TDF presented detectable HBV viremia and among them, LAM-resistant mutations were detected in five patients. Among the 34 patients with HBV detectable viremia, no TDF-resistant mutations were observed.

Forty-two patients (48. 9%) were HBeAg positive and 44 (51. 1%) were HBeAg negative. Baseline plasma HBV viral load was above 60 UI/mL among 34 (39. 5%) patients and below 60 UI/mL among 50 (58. 1%) out of 86 patients.

Among the 34 patients with detectable HBV DNA, HBV genotype distribution was: A-21 (61. 8%); D-5 (14. 7%); G-5 (14. 7%); F-3 (8. 8%). Genotypes B, C, and E were not detected. Electropherogram analysis did not show any evidence for the presence of mixed infections with different genotypes.

Presence of anti-HCV was observed in 20 out of 86 patients (23. 2%) and HCV-RNA was confirmed in 18 patients.

Only one (1. 1%) anti-HDV positive patient was identified. Current HDV infection was confirmed by PCR and the genotype of this case was determined by phylogenetic analysis of the sequence as HDV-1.

The mean CD4 T-cell count of the 86 patients was 545 (± 250) cells/μ1. Eighty-five percent of patients (73/86) had baseline HIV RNA < 400 copies/mL. A total of 37% of patients (32/86) had elevated ALT values. ALT levels were on average twice the upper normal limit.

In univariate analysis, presence of HBeAg was associated with HBV-DNA viral load > 60 IU/ml (p < 0. 001) and ALT level > 1. 5 times the upper normal limit (*p *= 0. 002) (Table 2). After multivariate analysis, presence of HBeAg remained associated with the same variables: HBV-DNA > 60 UI/mL (OR 5. 442891; 95% CI 1. 92026-15. 42763; *p = *0. 001); and ALT > 1. 5 × ULN (OR 3. 970025; 95% CI 1. 077474-14. 62782; *p = *0. 038).

**Table 2:** Predictors of HBeAg status among patients with HIV-HBV co-infection: Univariate analyses

	HBeAg Non-reagent	HBeAg Reagent	OR	95%CI	*p*
**Sex**					0. 59
**female**	2 (66. 67%)	1 (33. 33%)	1. 00		
**male**	42(50. 60%)	41 (49. 40%)	1. 95	0. 17-22. 37	
					
**Age**					0. 16
**< = 42 years**	18 (43. 9%)	23 (56. 1%)	1. 00		
**> 42 years**	26 (59. 09%)	18 (40. 91%)	0. 54	0. 22-1. 28	
**CD4**					
**< 350 cels/μ1**	12 (46. 15%)	14 (53. 85%)	1. 00		0. 54
**> 350 cels/μ1**	32 (53. 33%)	28 (46. 67%)	0. 75	0. 29-1. 88	
**Use of ARV**					0. 18
**no**	1(20%)	4 (80%)	1. 00		
**yes**	43 (53. 09%)	38 (46. 91%)	0. 22	0. 02-2. 06	
**HBV Genotype**					0. 68
**A**	5 (23. 8%)	16 (78. 1%)	0. 58	0. 1-3. 2	
**Non-A**	2 (19. 3%)	11 (84. 6)	1. 00		
**Previous use of LAM**					0. 64
**no**	3 (42. 8%)	4 (57. 1%)	1. 00		
**yes**	41(51. 9%)	38 (48. 10%)	0. 69	0. 14-3. 31	
**Previous use of TDF**					
**no**	22 (57. 8%)	16 (42. 11%)	1. 00		0. 26
**yes**	22 (45. 8%)	26 (54. 1%)	1. 62	0. 68-3. 83	
**Time of TDF use > 12 months**					0. 73
**no**	6 (50%)	6 (50%)	1. 00		
**yes**	16 (44. 4%)	20 (55. 5%)	1. 25	0. 33-4. 62	
**HBV-DNA**					**< 0. 001**
**< 60 UI/mL**	34(40. 9%)	15(18%)	1. 00		
**> 60 UI/mL**	08(9. 6%)	26(31. 3%)	21. 52	4. 58-100. 98	
**ALT Level**					**0. 002**
**Normal**	39(46. 9%)	24(28. 9%)	1. 00		
**> 1. 5 × ULN**	4(4. 8%)	16(19. 2)	6. 66	1. 99-22. 28	
**Compliance to therapy**					0. 26
**yes**	41 (53. 25%)	36 (46. 75%)	1. 00		

**no**	**3 (33. 3%)**	**6 (66. 6%)**	**2. 27**	**0. 53-9. 77**	

ULN-Upper Limit of Normality

For HBV viral load, the univariate analysis showed that HBV-DNA < 60 IU/ml was associated to previous exposure to either LAM or TDF (*p *= 0. 005), exposure to TDF (*p *= 0. 005), exposure to TDF for longer than 12 months (*p *= 0. 047), normal ALT levels (*p *= 0. 004), and compliance to therapy (*p *= 0. 033) (Table 3). After multivariate analysis, HBV-DNA < 60 IU/ml was only associated to use of TDF for longer than 12 months (*p *= 0. 047) (OR 0. 24; 95% CI 0. 059-0. 9794463; *p = *0. 047).

**Table 3:** Predictors of HBV-DNA viremia among patients with HIV-HBV co-infection: Univariate analyses

	Plasma HBV DNA < 60 UI/mL	Plasma HBV DNA > 60 UI/mL	OR	95% CI	*p*
**Sex**					0. 79
**female**	2 (66. 67%)	1(33. 33%)	1. 00		
**male**	48(59. 26%)	33(40. 74%)	1. 37	0. 11-15. 79	
**Age**					0. 41
**< = 42**	22(55%)	18(45%)	1. 00		
**> 42**	27(62. 79%)	16(37. 21%)	0. 72	0. 30-1. 74	
**CD4**					0. 47
**< 350 cels/μ1**	14 (53. 85%)	12(46. 15%)	1. 00		
**> 350 cels/μ1**	36 (62. 07%)	22 (37. 93%)	0. 71	0. 27-1. 81	
Under HAART*					***0. 005***
**yes**	50(63. 29%)	29(36. 71%)			
**no**	zero	05 (100%)			
**HBV Genotype**					
**A**	4 (20%)	16 (80%)	0. 36	0-2. 9	0. 63
**Non-A**	1 (8. 3%)	11 (91. 6%)	1. 00		
**Previous exposure to LAM**					0. 10
**no**	2(28. 57%)	5(71. 43%)	1. 00		
**yes**	48(62. 34%)	29(37. 66%)	0. 24	0. 04-1. 32	
**Under TDF**					***0. 005***
**no**	15(17. 8%)	21(25. 16%)	1. 00		
**yes**	35(41. 6%)	13(15. 4%)	0. 26	0. 10-0. 66	
**Use of TDF > 12 months**					***0. 047***
**no**	21(25%)	27(32. 1%)	1. 00		
**yes**	29(34. 5%)	7(8. 2%)	0. 24	0. 059-0. 97	
**ALT level**					***0. 004***
**Normal**	44(53. 6%)	19(23. 1%)	1. 00		
> 1. 5 ULN**	6(7. 3%)	13(15. 7%)	5. 01	1. 65-15. 17	
**Compliance with therapy**					***0. 033***
**no**	01(14. 29%)	06(85. 71%)	10. 5	1. 20-91. 71	
**yes**	49(63. 64%)	28(36. 36%)	1. 00		

Under HAART*- Previous use of either lamivudine (LAM) or tenofovir (TDF)

ULN**-Upper Limit of Normality

## Discussion

Our cross-sectional study evaluated the factors associated to HBeAg status and hepatitis B viremia in HIV-HBV co-infected patients in São Paulo, Brazil. Among 86 HIV/HBV co-infected patients with prior use of either LAM or TDF (or both), we showed that individuals receiving combination therapy with prolonged use of TDF were significantly more likely to have undetectable HBV DNA. We also found that presence of HBeAg was associated with detectable HBV viremia (HBV DNA > 60 IU/ml) and increased ALT levels. On the other hand, prolonged TDF use was not associated to HBeAg seroconversion.

In HBV mono-infected patients, successful HBV therapy can reduce the risk of developing complications related to liver disease [17,18]. Recent recommendations advocated undetectable HBV DNA as a treatment goal in HBV mono-infected patients and co-infected patients [19-23]. Reaching undetectable HBV DNA also minimizes the risk for developing drug-resistant HBV, which is one of the most undesirable complications of hepatitis B treatment [19].

The Brazilian AIDS treatment program provides free access to highly active antiretroviral therapy for all persons living with HIV/AIDS who seek treatment. The fact that LAM has been used at large in the country as anti-HIV therapy since the mid-1990s means that a great number of HIV patients in Brazil who are also infected with HBV, have received this drug for many years. This situation provides an opportunity to investigate the effects that this drug, and also TDF, have on HBV when used for long periods by HIV-HBV co-infected patients.

Most patients included in our study had previously used antiretroviral therapy, which included LAM (91. 8%). All patients receiving TDF had previous use of LAM. Among our group of patients, time of exposure to TDF and LAM varied from 4 to 21 and 7 to 144 months, respectively. Nevertheless, only the prolonged use of TDF (more than 12 months) was associated with undetectable HBV viral load.

In patients already using LAM, the use of TDF can eventually lead to a slower decrease in HBV viremia [24-27]. This probably explains why we only observed association between undetectable HBV viremia and prolonged TDF use. Among our patients, the use of TDF was typically associated with undetectable or low HBV-DNA level. Our results are in accordance with previous studies that addressed the same question [27-29].

Current guidelines recommend the use of TDF and emtricitabine (FTC), or TDF and LAM, as the nucleos(t)ide reverse transcriptase inhibitor (NRTI) backbone of antiretroviral regimens in HIV/HBV co-infected individuals. The benefit of a combination therapy in which TDF is included over its use as a monotherapy for HBV has not been definitively demonstrated in any patient population, including HBV/HIV co-infected patients. More studies are needed to determine whether TDF maintains its efficacy for longer periods.

We were able to determine the HBV genotypes in 34 of the patients with detectable HBV viremia. Among them, the HBV genotype distribution was as follows: A-21 (61. 8%); D-5 (14. 7%); G-5 (14. 7%); F-3 (8. 8%). Genotypes B, C, and E were not detected. Genotypes B and C are typical from Asian descendants living in Brazil although we do not have any patient with this ancestry in our study, while genotype E has only been found in Brazil in African immigrants [30].

Our findings suggest that the risk for virological non-response is not associated to HBV genotype, as previously suggested by other studies [31]. This may be explained by the fact that data on HBV genotype distribution were not available before patients started the therapy. Therefore the genotype distribution reflects that among patients under the use of LAM or TDF and presenting detectable viremia, which may not reflect the genotype distribution of all patients included in our study.

In our population, five cases of genotype G were identified. This genotype is uncommon in Brazil

The presence of genotype G has been associated by some authors with a sexual transmission among males [32]. Indeed, the five patients in our study carrying this genotype were homosexual males. Additional studies with larger sample populations that analyze HBV genotypes in individuals with different sexual behaviors will be enlightening.

Among the 86 HBsAg-positive patients, 42 (48. 8%) were HBeAg positive and 44 (51. 1%) were HBeA negative. These results agree with other studies also describing a predominance of cases of patients with HBeAg positivity among HIV-HBV co-infected patients [29,31,33,34]. Because only a few genotypes were not A genotypes we were unable to analyze the distribution of HBeAg seropositivity in the sera of individuals carrying different HBV genotypes.

Our data showed an association between HBeAg positivity and HBV-DNA viremia. Several studies have already addressed this question in HIV-HBV co-infected patients. Our study confirm this data [34,35].

In HBeAg-positive chronic hepatitis B, HBeAg and HBV DNA levels are supposed to follow parallel changes in their levels as they both indicate viral replication. It is noteworthy that despite the fact that in our study prolonged use of TDF was associated with undetectable HBV DNA, HBeAg was still detectable in most patients. This finding reflects the initial effects of TDF in the replicating viral DNA. Its efficacy in decreasing HBeAg and even HBsAg levels takes longer, as it reflects a decrease in the translation of viral mRNAs that depends on decreasing levels of the viral DNA in the hepatocyte nuclei, especially the cccDNA.

Among mono-infected HBeAg-positive patients, HBe seroconversion rates are of the order of 20%, after one year of TDF use. Among co-infected patients on TDF use, higher rates of seroconversion have been shown in some cohorts (33%-36%), but only in antiretroviral-naïve individuals and not when TDF was introduced as a second-line agent. This might be a consequence of immune reconstitution and acquisition of either enhanced pathogen-specific innate or adaptive immune responses to HAART [36-39].

Most of our patients had previous use of TDF as a second-line therapy. Therefore it is reasonable to suppose that for this group of patients a longer follow-up would be necessary in order to observe HBeAg seroconversion. We have not applied in this study quantitative assays for HBe or HBsAg that would allow us to follow their levels during the treatment.

In our study, HBeAg positivity was also associated with ALT levels 1. 5 times the upper limit of normality. In patients with HIV-HBV co-infection, little is known about the association between HBeAg positivity and ALT levels. Previous studies, mostly in the pre-HAART era, have suggested that liver damage (assessed by ALT levels) is less severe in persons with HIV infection and active HBV replication [40,41]. Nevertheless, the natural histories of HIV and HBV infections have been touched by the advent of HAART.

These multidrug regimens changed the course of liver disease in co-infected patients. The primary mechanism of HBV-associated liver damage is cell-mediated immune response. In the present study, most patients were on HAART, which most likely resulted in at least partial immunological reconstitution. At baseline, the mean CD4+ T-cell count of the patients was 545 cells/mm^3^. Most of the patients (91. 8%) had previous experience with LAM and resistance mutations were frequently found among patients. We wonder whether the rise in ALT levels is linked to the partially reconstituted immune status of these patients.

ALT is considered a marker of hepatic necrosis and inflammation. As stated above, our study had a cross-sectional design, precluding analysis of serially tested specimens and precluding determination of the evolution of ALT variations over time. It would also be reasonable to suppose that for this group of patients a longer follow-up would be necessary in order to observe ALT normalization, assuming that patients would be adherent to treatment. Rises in ALT in patients with HIV have multiple etiologies, including antiretroviral therapy, alcohol and other drug use, as well as opportunistic infections. No particular cause has been identified in the present study although none of these etiologies can be ruled out either.

It is noteworthy that the presence of anti-HCV was also observed in 20 out of 86 patients and HCV-RNA was confirmed in 18 patients. HCV co-infection may also have contributed to elevation in ALT observed in this group of patients.

Furthermore, hepatitis delta virus (HDV) was found in only one out of 86 patients analyzed. The prevalence of anti-delta antibodies in HIV patients with detectable HBsAg ranges from 15% to 50%, depending on geographical region and risk group category [42]. In Brazil, HDV is endemic in the northern part of the country (Amazon Basin) and is only exceptionally found outside this area [43]. This fact probably explains why only one patient with hepatitis delta was identified. It is interesting to note that the genotype of this case was determined by phylogenetic analysis of the sequence as HDV-1.

This study has some limitations. First, it has a cross-sectional design, precluding analysis of serially tested specimens and precluding determination of the evolution of serological markers (in particular to follow the evolution of HBeAg status) or HBV viral load before antiviral therapy usage. HAART status and HBV characteristics were assessed at a single time point (at baseline) and data associated to HBV and HIV prior to HAART initiation were not uniformly acquired. Thus, we cannot rule out that the associations we observed were already present before these drugs were introduced. Second, compliance with therapy was not confirmed. Finally, we believe that follow-up of this cohort will be needed to determine if the associations observed in our study regarding HBeAg status and HBV viremia will remain over time and to determine factors that might be associated with the development of TDF resistance.

## Conclusions

In summary, prolonged use of TDF was able to control HBV replication in most HIV-HBV co-infected patients. The prescription of HAART containing anti-HBV drugs, particularly TDF, had a positive impact on the virological outcome of hepatitis B in HIV-HBV co-infected patients, regardless of previous LAM treatment.

## Competing interests

The authors declare that they have no competing interests.

### Financial Competing interests

In the past five years the authors did not receive any reimbursements, fees, funding, or salary from any organization that may in any way gain or lose financially from the publication of this manuscript, either now or in the future. The authors also declare that there is no organization financing this manuscript (including the article-processing charge). The authors declare that do not hold any stocks or shares in an organization that may in any way gain or lose financially from the publication of this manuscript, either now or in the future. The authors declare that they are not currently applying for any patents relating to the content of the manuscript.

### Non-financial Competing interests

The authors declare that there are no non-financial competing interests (political, personal, religious, ideological, academic, intellectual, commercial, or any other) to declare in relation to this manuscript.

## Authors' contributions

MCMC and DEU designed the study. ACS, JRRP, and MSGG did most of the laboratory work. CFG, LGM, AGL, and MHS provided and checked the clinical data for patients. MCMC, CFG, LGM, AGL, and MHS also participated in data collection.

MCMC and JRRP wrote the manuscript. RJG participated in the design of the study and performed the statistical analysis. All authors reviewed the draft and approved the final version.

## Pre-publication history

The pre-publication history for this paper can be accessed here:

http://www.biomedcentral.com/1471-2334/11/247/prepub
